# Vitamin E as a Treatment for Nonalcoholic Fatty Liver Disease: Reality or Myth?

**DOI:** 10.3390/antiox7010012

**Published:** 2018-01-16

**Authors:** Hamza El Hadi, Roberto Vettor, Marco Rossato

**Affiliations:** Internal Medicine 3, Department of Medicine—DIMED, University of Padova, Via Giustiniani 2, 35128 Padova, Italy; dr.hamza.elhadi@gmail.com (H.E.H.); roberto.vettor@unipd.it (R.V.)

**Keywords:** vitamin E, nonalcoholic fatty liver disease, obesity, oxidative stress

## Abstract

Obesity is one of the major epidemics of this millennium, and its incidence is growing worldwide. Following the epidemics of obesity, nonalcoholic fatty liver disease (NAFLD) has become a disease of increasing prevalence and a leading cause of morbidity and mortality closely related to cardiovascular disease, malignancies, and cirrhosis. It is believed that oxidative stress is a main player in the development and progression of NAFLD. Currently, a pharmacological approach has become necessary in NAFLD because of a failure to modify lifestyle and dietary habits in most patients. Vitamin E is a potent antioxidant that has been shown to reduce oxidative stress in NAFLD. This review summarizes the biological activities of vitamin E, with a primary focus on its therapeutic efficacy in NAFLD.

## 1. Introduction

Nonalcoholic fatty liver disease (NAFLD) is defined as the accumulation of excessive fat in the liver, as demonstrated by imaging or by histology, in the setting of no significant alcohol consumptionand the absence of any secondary cause [1].

NAFLD encompasses a broad pathological spectrum of phenotypes ranging from isolated hepatic steatosis (IHS) to nonalcoholic steatohepatitis (NASH)—the progressive form of fatty liver disease associated with inflammation and cellular injury, which can lead to NASH-related cirrhosis and hepatocellular carcinoma [2]. This pathology is now regarded as a leading cause of chronic liver diseases and liver transplantation in most countries [3]. In addition, NAFLD has been also linked to extra-hepatic morbidity, including systemic metabolic complications, chronic kidney and cardiovascular disease, and malignancies, which all contribute to a higher mortality observed in NASH patients [4].

NAFLD is strongly associated with obesity and related metabolic disorders such as insulin resistance and dyslipidemia. In the last decades, adult and childhood obesity has reached epidemic levels, and as a consequence the global prevalence of NAFLD has increased significantly. According to a recent report, prevalence estimates in the general population of Europe and the Middle East are 20–30%, with higher prevalence in Western countries’ populations with obesity or diabetes (75%) and with morbid obesity (90–95%) [5].

It is acknowledged that vitamin E is the major lipid-soluble chain-breaking antioxidant found in the human body. In addition to its anti-oxidative properties, molecules of the vitamin E family exertanti-atherogenicand anti-inflammatory activities [6]. Although the pathogenesis of NAFLD and its progression to fibrosis needs to be fully clarified, it is believed that oxidative stress plays a crucial role in producing the lethal hepatocyte injury associated with NAFLD.

Therefore, by targeting oxidative stress components, vitamin E appears as a promising therapeutic approach in NASH patients.

The present review briefly discusses the biological activities of vitamin E, focusing on its potential as a treatment for NAFLD/NASH. To this aim, we also highlight the role of oxidative stress in the pathogenesis of NAFLD.

## 2. Vitamin E: Brief Overview of Structure, Metabolism and Function

### 2.1. Structural Perspectives

**Figure 1:**
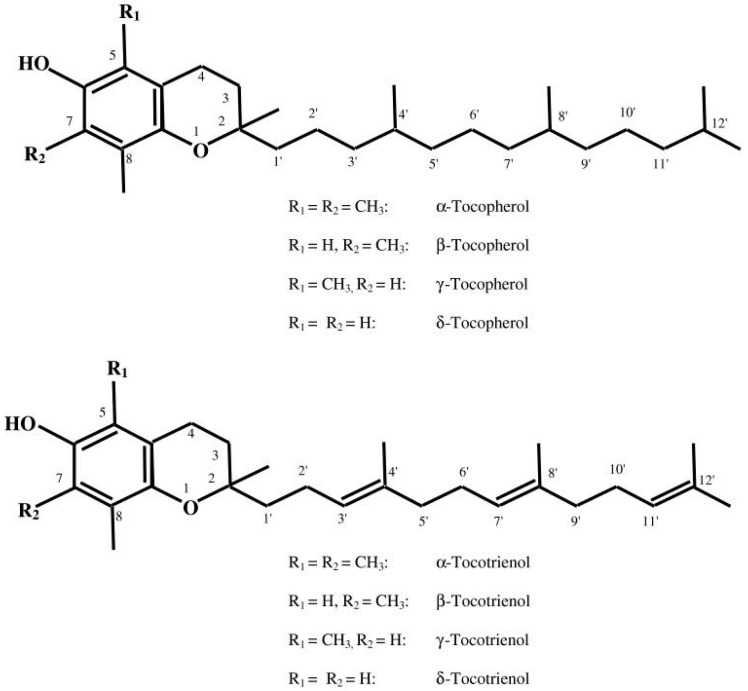
Structure of tocopherols and tocotrienols (with permission from reference [8]).

The discovery of vitamin E dates back to 1922 due to the observations of Herbert Evans and his associate Bishop who isolated an as-yet uncharacterized fat-soluble compound from green leafy vegetables which is required for reproduction in rats [7]. Upon isolating this dietary compound, it was termed as tocopherol (Greek: tocos—child birth; pheros—to bear; ol—alcohol). Today the term “vitamin E” encompasses a group of eight lipophilic molecules that are synthesized by plants starting from homogentisic acid. It includes four tocopherols and four tocotrienols. Tocopherols and tocotrienols are subdivided into alpha (α), beta (β), gamma (γ), and delta (δ) forms based on the methyl and hydroxyl substitution in their phenolic rings [8] (Figure 1). The tocopherols are saturated forms of vitamin E, whereas the tocotrienols are unsaturated and possess an isoprenoid side chain [8]. α-Tocopherol is considered the most abundant form in nature, and is consequently the most widely studied. Common food oils including corn, peanut, and soybean oil contain largely α-tocopherol. In contrast, tocotrienols are relatively rarer in food sources and prevail in rice bran, barley, oats, and palm oil [9].

Currently, synthetic forms of vitamin E consist mainly of α-tocopherol, whichwas first synthesized in 1938 [10]. Unlike naturally-occurring d-RRR-α-tocopherol, synthesized α-tocopherol consists of a racemic mixture of eight stereoisomers named the all-racemic or (2RS, 4’RS, 8’RS) product [11].

### 2.2. Insight into Metabolism

Like other fat-soluble vitamins, the bioavailability of vitamin E depends on pancreatic function, biliary secretion, micellar formation, and penetration across intestinal membranes. After being cleaved by the enzyme esterase located in the stomach lining and partly processed enzymatically in the stomach by gastric lipase, vitamin E isoforms reach the basolateral side of enterocytes [12,13]. In the intestinal lumen, dietary tocopherols and tocotrienols appear to be similarly absorbed along with dietary fat, and are secreted in chylomicron particles together with triacylglycerol, phospholipids, and cholesterol [12,13]. The chylomicron-bound vitamin E isoforms are then transferred to peripheral tissues such as adipose tissues, bones, brain, lung, muscle, and skin via the lymphatic system. During their transport, chylomicrons are catabolized in the circulation by the endothelial-bound enzyme, which hydrolyzes triglycerides, releasing free fatty acids (FFAs) [12,13]. The resulting chylomicron remnants containing absorbed vitamin E are subsequently taken up by the liver, probably by a receptor-mediated process, and then preferentially incorporated within very low density lipoprotein (VLDL) and high-density lipoprotein (HDL) into the bloodstream [12]. After hepatic uptake, the α-tocopherol form of vitamin E is preferentially re-excreted into the circulation. α-Tocopherol transfer protein (α-TTP), a small cytoplasmic hepatic protein with differential affinity for various vitamin E forms, is responsible for the biodiscriminating process underlying the selective resecretion of α-tocopherol from the liver into plasma [14].

While α-TTP has a high affinity for α-tocopherol (100%), it has a lower affinity for other vitamin E isoforms: approximately 50% for β-tocopherol, 10–30% for γ-tocopherol, and 1% for δ-tocopherol [12]. In the liver, isoforms not bound to α-TTP will be susceptible to catabolization via cytochrome P450 (CYP4F2)-initiated ω-hydroxylation and oxidation by ω-hydroxylase, and thus vitamin E isoforms are metabolized to carboxychromanols, hydroxycarboxychromanol, and carboxyethylhydroxychroman derivatives [15]. Besides catabolism, it was estimated that metabolized vitamin E isoforms are also discarded via biliary excretion [16].

### 2.3. Biological Activity of Vitamin E

#### 2.3.1. Antioxidant Activity

Oxidative stress is defined as the imbalance between the generation of reactive species and antioxidant defense, and leads to the damage of DNA and disturbances in cellular biology [17]. Vitamin E is widely accepted as one of the most potent antioxidants in nature [18]. The antioxidant property is attributed to the hydroxyl group from the aromatic ring of tocochromanols, which donates hydrogen to neutralize free radicals or reactive oxygen species (ROS). The antioxidant activity of α-, β-, γ-isoforms of tocotrienols and tocopherols is similar, but the γ-isoform showed weaker activity when tested in pyrogallolsulfonphthalein and 2,7-dichlorodihydrofluorescein diacetate methods [18,19].

In the same context, the antioxidant efficacy of vitamin E on reactive nitrogen species (RNS) has been gaining more attention recently. RNS include nitric oxide (NO), nitrogen dioxide (NO_2_), and peroxynitrite (ONOO ^–^) [20,21,22].

On the other hand, in vitro studies have shown that vitamin E can alternatively switch to a pro-oxidant action under certain circumstances, such as a constant low-level flux of initiator free radicals and the absence of co-antioxidants as such as vitamin C [23]. In addition, current evidence from in vivo studies showed that vitamin E may produce pro-oxidant effects at high doses [24] or in cigarette smokers consuming a high polyunsaturated fat diet [25].

#### 2.3.2. Beyond Vitamin E Antioxidant Activity

Vitamin E biological activity is not limited to antioxidant properties. In fact, vitamin E forms are involved in the regulation of inflammatory response, gene expression, membrane-bound enzymes, modulation of cellular signaling, and cell proliferation.

Over the last two decades, vitamin E has been shown to have direct and indirect effects on several enzymes involved in signal transduction, such as protein kinase C (PKC), protein phosphatase 2A (PP2A), protein tyrosine phosphatase (PTP), protein tyrosine kinase (PTK), diacylglycerol kinase (DAGK), 5-, 12- and 15-lipoxygenases (5-, 12-, and 15-LOX), phospholipase A2 (PLA2), cyclooxygenase-2 (COX-2), and the mitogen activated protein kinase (MAPK) signal transduction pathway [26,27].

The first evidence that vitamin E can modulate enzymes involved in signal transduction came from studies with PKC when α-tocopherol exerted an inhibitory effect unrelated to antioxidant action [28]. PKC inhibition by α-tocopherol is mainly correlated with the reduction of cell proliferation in many different cell types, including vascular smooth muscle cells, monocytes/macrophages, neutrophils, fibroblasts, mesangial cells, as well as various cancer cell lines [29]. In addition to interference with cell proliferation, the inhibition of PKC by α-tocopherol inhibited NADPH-oxidase assembly in monocytes, leading to lower superoxide production [30]. Additionally, PKC inhibition mediated by α-tocopherol and not by β-tocopherol suppressed endothelin secretion in endothelial cells [31].

Another evidence of a non-antioxidant function of α-tocopherol is related to its role in regulating the expression of specific genes not only coupled to oxidative stress but also involved in cholesterol homeostasis, inflammatory pathways, and cellular trafficking [32,33]. These genes include those encoding for proteins involved in inflammation and cell adhesion (such as E-selectin, intercellular adhesion molecule-1, vascular cell adhesion molecule [VCAM]-1, integrin, interleukin [IL]-1b, IL-2, IL-4, and transforming growth factor [TGF]-β), extracellular matrix formation and degradation (tropomyosin, glycoprotein IIb, collagen A1, matrix metalloproteinase [MMP]-1, MMP-19 and connective tissue growth factor), cell cycle regulation (cyclin D1, cyclin E1, and p27), transcriptional control lipoprotein (peroxisome proliferator-activated receptor [PPAR]-γ), receptors (CD36, scavenger receptor class B type 1, low density lipoprotein receptor), and metabolism (Cytochrome P450 3A4 [CYP3A4] and HMG-CoA reductase) [32,33].

## 3. Vitamin E and NAFLD

### 3.1. NAFLD/NASH Pathogenesis

**Figure 2:**
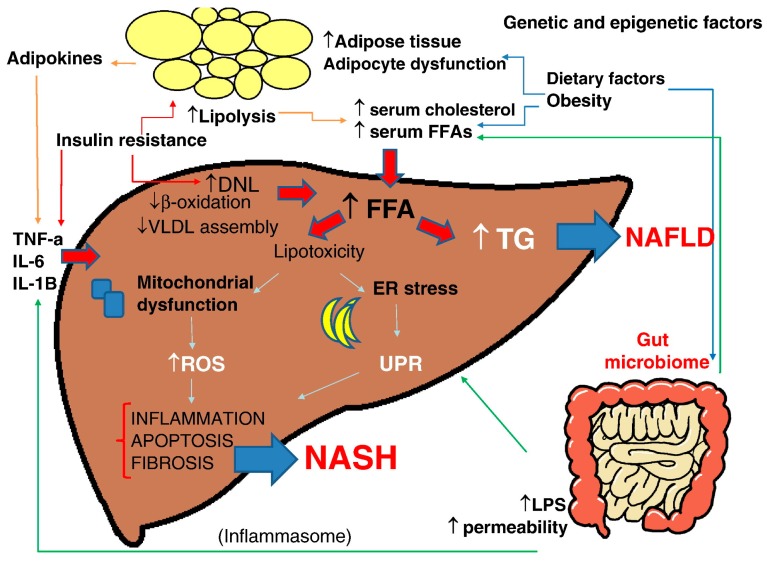
Multiple parallel-hit hypothesis of nonalcoholic fatty liver disease (NAFLD). Obesity together with dietary habits and environmental factors can lead to raised serum levels of free fatty acids (FFAs) and cholesterol, development of insulin resistance, adipocyte proliferation, and dysfunction in the intestinal microbiome. Insulin resistance acts on adipose tissue, worsening adipocyte dysfunction, induces hepatic de novo lipogenesis (DNL) and release of proinflammatory adipokines such as interleukin (IL)-6, IL-1β, and tumor necrosis factor (TNF)-α, which also exacerbates the insulin resistance state. The increased hepatic FFAs flux which derives from the above processes and from an altered activity of the gut microbiome leads to accumulation of triglycerides (TGs) and “toxic”levels of FFAs, free cholesterol, and other lipid metabolites which cause mitochondrial dysfunction, oxidative stress with the production of reactive oxygen species (ROS), and endoplasmic reticulum (ER) stress with the activation of the unfolded protein response (UPR), all leading to hepatic inflammation and fibrogenesis (nonalcoholic steatohepatitis, NASH). Increased intestinal permeability of gut-derived microbial products such as lipopolysaccharides (LPS) contributes to the activation of the inflammasome, ER stress, and activation of inflammatory cascades. Epigenetic factors are also involved in progression to NASH or persistence in a stable stage of disease (with permission from [34]). VLDL: very low density lipoprotein.

NAFLD is a complex disease trait where inter-patient genetic and epigenetic variations and environmental factors are combined to define development and disease progression [34] (Figure 2).

The generally accepted dogma in the pathogenesis of NAFLD is that insulin resistance—commonly associated with obesity—leads to the hepatic accumulation of triglycerides, a process that usually results from increased FFAs flux from adipose tissue to the liver, dietary fat via chylomicron metabolism, and increased de novo lipogenesis [35,36,37]. Obesity generates a state of low-grade inflammation characterized by the accumulation of immune cells in adipose tissue (particularly macrophages), and the production of proinflammatory cytokines by adipocytes contributes to the development of systemic and hepatic insulin resistance [35,36,37]. However, multiple pathways (multiple hits) are involved in the development of NASH and fibrosis. “Hits” that may contribute include oxidative stress, endotoxins, changes in the gut–liver axis, and mitochondrial dysfunction [34].

Oxidative stress and mitochondrial dysfunction were proposed as main triggers for the progression of steatosis to steatohepatitis [38]. FFAs catabolism in the liver takes place mainly via mitochondrial β-oxidation—a process that can lead to the generation of ROS, including superoxide, hydrogen peroxide, and hydroxyl radicals, in the case of increased FFAs delivery [38].

In the same context, impaired mitochondrial activity in NASH patients due to reduced enzymatic activities of mitochondrial electron transport chain and excessive in FA oxidation results in hepatic ATP depletion and may cause structural mitochondrial abnormalities consisting of enlargement (megamitochondria), paracrystalline inclusions, and loss of cristae [39,40].

In NAFLD, enhanced cytochrome P450 2E1 (CYP2E1) expression and activity seem to be an important source of ROS which trigger oxidative stress and perpetuate the hepatic mitochondrial dysfunction [41]. Moreover, it has been reported that upregulated microsomal CYP4A enzymes ω-hydroxylate fatty acids into dicarboxylic acids that are then preferentially oxidized by peroxisomes, thus promoting ROS production in NAFLD [42]. The ablation of a homolog of human CYP4A gene (CYP4A14) in animal models of steatohepatitis has shown to attenuate hepatic inflammation and fibrosis [43]. The abundant production of ROS induces the peroxidation of hepatic triglycerides with the release of reactive aldehydes such as 4-hydroxynonenal (4-HNE) and malondialdehyde (MDA) which can damage mitochondrial components [44]. 

Accumulating data have implicated the disruption of endoplasmic reticulum (ER) homeostasis (i.e., ER stress) in the development of NASH [45]. Factors that disturb ER folding capacity (e.g., excessive protein synthesis, mitochondrial dysfunction, oxidative stress) will lead to the activation of a physiologic mechanism called the “unfolded protein response” (UPR) in hepatocytes. This adaptive mechanism aims to increase the folding capacity of the ER, thus bringing the organelle and the cell into a state of equilibrium. When the activation of the UPR fails to promote cell survival, the cell is taken down the pro-apoptotic ER stress response pathway, which can ultimately lead to apoptotic cell death, inflammation, and/or fat accumulation [45]. 

Several studies have suggested a role of gut microbiome in NASH pathogenesis [46]. Intestinal barrier alterations cause increased intestinal permeability of bacteria, viruses, or microbial products such as lipopolysaccharide (LPS). These pathogens are recognized through specialized recognition receptors that include toll-like receptors (TLRs) and inflammasomes, inducing a signaling cascade leading to the production of inflammatory cytokines [46].

### 3.2. Vitamin E and NAFLD: Experimental Studies

The methionine and choline deficient (MCD) diet is a widely employed diet in NASH animal studies. The MCD diet is high in sucrose and fat (40% sucrose, 10% fat), and is deficient in methionine and choline, which are essential for hepatic beta-oxidation and the production of VLDL [47].

A trial carried out with rats fed with an MCD-diet showed that vitamin E significantly reduces the oxidative stress. In the control group, the authors observed a depletion in the liver glutathione stores and a notable increase in hepatic fibrosis, whereas vitamin E supplementation repleted hepatic glutathione, reduced steatosis, inflammation, hepatic stellate cell activation, and collagen mRNA expression, and ameliorated fibrosis [48].

In another animal NASH model, the combination of MCD diet and vitamin E significantly lowered serum transaminase levels and ameliorated hepatic steatosis and necroinflammation. These effects were associated with suppressed expression of the fibrotic genes TGF-β and MMP-2, inflammatory factor COX-2, and pro-apoptotic genes (Bax). In addition, vitamin E enhanced the activity of hepatic superoxide dismutase (SOD) and inhibited that of nuclear factor kappa B (NFkB) [49].

Similar effects were reported in arecent mouse model for NAFLD in which vitamin E therapy after partial hepatectomy significantly reduced the oxidative stress level and attenuated the progression of NAFLD [50].

In an obese (ob/ob) mouse model, α- or γ-tocopherol exerted a hepatoprotective role in alipopolysaccharide-induced NASH, as shown by suppressing hepatic malondialdehyde (MDA), tumor necrosis factor-α, and serum alanine aminotransferase levels [51].

Moreover, chickens fed ahigh-oxidant diet with the supplementation of vitamin E were able to normalize elevated hepatic transaminase levels [52]. In addition to the role of being an antioxidant, some studies have also proposed that vitamin E improves the liver integrity by down-regulating hepatic cluster of differentiation 36 protein (CD36)—a membrane transporter responsible for the uptake of fatty acids into the liver [53].

### 3.3. Vitamin E and NAFLD: Human Studies

Vitamin E has been used in monotherapy or with other agents in multiple clinical trials to treat NAFLD or NASH, with reported improvement in liver biochemistries and histology [54,55,56,57,58,59,60,61,62,63,64,65,66,67,68]. These trials varied in duration (24 weeks to >2 years) and dose (100–1200 IU/day) of vitamin E used. Long-term studies (≥2 years) are summarized in Table 1.

Treatment with vitamin E combined with vitamin C and atorvastatin was demonstrated to be effective in reducing the odds of having hepatic steatosis in individuals with computed tomography (CT)-diagnosed NAFLD after 4 years of active therapy [63].

The combination of ursodeoxycholic acid (UDCA) with vitamin E has been evaluated in a small sample size compared with UDCA alone or placebo. Improvement in steatosis and transaminases level were observed only in the group who received combination therapy with UDCA and vitamin E [64]. By evaluating the long-term (>2 years) efficacy of a similar combination (UDCA with vitamin E) in patients with NASH, Piettu et al.demonstrated an improvement in histological lesions in the majority of patients [62].

Nobili et al.studied 90 children with NAFLD who were given calorie-restricted diet and exercise. Patients were randomized to treatment with vitamin E (600 IU/day) in combination with ascorbic acid (500 mg/daily; *n* = 45) or placebo (*n* = 45) [65]. At the end of 24 months, both groups had significant improvement in steatosis, lobular inflammation, hepatocyte ballooning. However, the addition of α-tocopherol and ascorbic acid was not associated with a greater histological or biochemical improvement as compared to placebo [65,66].

Clinical trials with vitamin E supplementation in NASH patients yielded promising results. In the PIVENS (Pioglitazone, Vitamin E or Placebo for Nonalcoholic Steatohepatitis) trial, vitamin E was evaluated as a treatment for NASH. The rate of achievement of the primary outcome was higher in patients treated with high-dose vitamin E (800 IU/day) for 96 weeks compared to placebo (43% vs. 19%, *p* = 0.001), while pioglitazone did not reach statistical significance. The histological analysis showed a reduction in hepatocyte ballooning (50% vs. 29%, *p* = 0.005) and lobular inflammation (54% vs. 35%, *p* = 0.02), thus reflecting its expected effect as an antioxidant leading to a decrease of oxidative stress-mediated injury. Interestingly, it significantly reduced liver steatosis and alanine aminotransferase (ALT), but had no significant changes on fibrosis [60].

Following those studies, Lavine et al. conducted a multicenter, double-blind, double-placebo, randomized clinical trial in pediatric patients. The TONIC (Treatment of NAFLD in Children) trial involved 173 children and adolescents witha mean age of 13 years that received metformin (500 mg twice daily), vitamin E (400 IU twice daily), or placebo twice daily for 96 weeks. The primary outcome was sustained reduction in ALT level (defined as ≤50% baseline or ≤40 U/L from 48 weeks to 96 weeks of treatment). The only histologic feature of NASH that improved after treatment with both medications was the hepatocellular ballooning. Disappointingly, neither vitamin E nor metformin were superior to placebo in achieving sustained ALT reduction or in improving steatosis, lobular inflammation, or fibrosis scores [61].

In the light of these observations, today the European Association for the Study of the Liver (EASL) and the American Association for the Study of Liver Diseases (AASLD) (Alexandria, VA, USA) guidelines consider vitamin E as a potential short-term treatment for non-diabetic adults with biopsy-proven NASH [69]. Until further data supporting its effectiveness is available, vitamin E is not recommended to treat NASH in diabetic patients, NAFLD without liver biopsy, NASH cirrhosis, or cryptogenic cirrhosis [1,69].

### 3.4. Vitamin E: Safety Concerns

Long-term safety should be carefully discussed with NASH patients before starting therapy with vitamin E.A meta-analysis has suggested that high-dosage (≥400 IU/day) vitamin E supplements may increase all-cause mortality [70]. However, this meta-analysis has been criticized because several studies with low mortality were excluded and concomitant vitamin A and the administration of other drugs as well as common factors such as smoking were not considered. Another meta-analysis where new clinical trials and updated results of mortality were included suggested that the higher mortality can be explained by a higher proportion of male patients that were included in these trials and not due to the higher dose of vitamin E supplementation [71]. On the other hand, large meta-analysis that included 57 trials showed that vitamin E supplementation appears to have no effect on all-cause mortality at doses up to 5500 IU/day [72].

Finally, a meta-analysis investigating the effect of vitamin E on the incidence of stroke reported an increase in the relative risk of hemorrhagic stroke by 22%, while the risk of ischemic stroke was reduced by 10% [73]. Another concern about vitamin E use is related to its association with a modest increase in the risk of prostate cancer [74].

Thus, patients chosen for treatment with vitamin E should be aware of these risks or be considered for alternative treatments.

## 4. Conclusions

Despite its high prevalence and the intensive research in the field, the treatment of NAFLD remains an unmet medical need. To date, no definite pharmacological treatment has been approved for NAFLD, and patients are often advised to engage in physical activity and lose weight, which is difficult to achieve and more difficult to maintain. However, policies to promote physical activity and the management of associated comorbidities (i.e., obesity and metabolic syndrome components) are expected to decrease both hepatic and cardiovascular-related morbidity and mortality in NASH patients.

Clinical trials for NAFLD or NASH showed a modest improvement in liver biochemistries and histology induced by vitamin E administration. However, some limitations of these trials should be noted. The duration of therapy may be not long enough to detect long-term histological change complications. Moreover, the number of randomized trials included was limited, and the number of participants in the trials was low. An additional reason for the lack of efficacy of vitamin E could be related to the use of oral preparations of varied dosage, which may not always guarantee an adequate bioavailability in patients with liver disease.

In this context, further monotherapy clinical trials and pharmacological evaluations are still needed to elucidate the underlying molecular mechanisms of prevention or therapy and possible adverse outcomes. This may also help to identify the optimal daily intake of vitamin E in both pediatric and adult patients.

In addition, more research is needed to seek novel biological activities of vitamin Emetabolites in the liver of NAFLD patients that may differ from those of parent compounds.

Finally, a better understanding of the pathophysiology of NAFLD/NASH will provide the opportunity to create trials of combination therapies that achieve high rates of therapeutic responses.

## Figures and Tables

**Table 1:** Effects of Vitamin E administration in patients with NAFLD.

Author	Study Design	Duration	Vitamin E Dosage	Effect of Vitamin E on NAFLD/NASH Histology and Biochemistry Compared to Placebo				
				ALT	Steatosis	Inflammation	Hepatocytes Ballooning	Fibrosis
Dufour et al., 2006 [64]	48 NASH patients randomized to vitamin E plus UDCA, UDCA versus placebo	2 years	400 IU twice daily	↓	↓	ns	NA	ns
Nobili et al., 2008 [66]	53 NAFLD patients randomized to vitamin E plus ascorbic and life style intervention versus placebo	2 years	600 IU/day	ns	ns	ns	ns	ns
Sanyal et al., 2010 [60]	247 NASH patients randomized to vitamin E, pioglitazone versus placebo	96 weeks	800 IU daily	↓		↓	↓	ns
Foster et al., 2011 [63]	1005 randomized to vitamin E combined with ascorbic acid and atorvastatin versus placebo. At baseline 80 had CT-diagnosed NAFLD	4 years	1000 IU daily	ns	↓ (Based on abdominal CT scan)	NA	NA	NA
Lavine et al., 2011 [61]	173 NAFLD patients randomized to vitamin E, metformin versus placebo	96 weeks	400 IU twice daily	ns	ns	ns	↓	ns
Pietu et al., 2012 [62]	101 patients treated with a combination of vitamin E with UDCA. 10 patients had a second liver biopsy during follow-up	4 years	500 IU daily	↓	↓ 3/10 ↑ 1/10 patients	↓ 3/10 ↑ 2/10 patients	↓ 3/10 ↑ 1/10patients	↓ 4/10 ↑ 1/10 patients

ALT: alanine aminotransferase; CT: computed tomography; NAFLD: nonalcoholic fatty liver disease; NASH: nonalcoholic steatohepatitis; ns: no significant statistical difference versus placebo; NA: not available; UDCA: ursodeoxycholic acid; ↑: worsening; ↓: improvement.
